# The Diet during School Life

**Published:** 1907-08-10

**Authors:** 


### Diseases of Children.

### THE DIET DURING SCHOOL LIFE.

Children are much better fed during school life than in former days, but there is still room for improvement. It is not always realised that the child requires relatively a greater supply of food than the adult. In addition to providing for the maintenance of the body heat, the energy of all vital processes, and the wear and tear of the body cells, the food must also provide for growth. Nitrogenous foods are especially necessary, for every cell is laid down in protoplasm.

The basis of the diet should be sufficiency in quantity, satisfactory quality, and variety. Deficiency is most likely to occur in the more expensive articles of diet, such as animal proteids, fats, and vegetables. Sometimes the right amount of proteid food is given, but it is not properly distributed among the different meals. An examination of the waste tub will show whether the diet is sufficient in quantity, provided that lack of variety and defective quality do not lead to refusal of food.

### Quantity and Quality.

In the matter of quality a just medium must be aimed at, for it is as unreasonable to expect the child to be fed on the best of all possible foods as it is unjustifiable to give that which is cheap and indigestible. Much can be done to render even coarse meats and other foods suitable by proper cooking. Good cooking is essential to the proper utilisation of food, to good digestion, to the encouragement of appetite, and the prevention of waste. It is also of importance that food should be well carved and served hot. Though a hungry child may eat almost anything, the appetite of many is spoilt by half-cold greasy gravy, cold potatoes, dirty plates, and soiled napery. Due attention to such matters is, moreover, a good object lesson to the child, for it teaches cleanliness, the importance of attention to detail, and that work must not be half done. Variety in meals should receive special attention. Above all, it is imperative that food should be fresh, meat not tainted, bread and milk clean and not sour. With a little care it is easy to give varied meals for a month in succession. Children should rise at 7 a.m. in summer and 7.30 a.m. in winter, be allowed twenty minutes to dress, and then given milk or cocoa, with biscuit or bread and butter. After that they may go to morning chapel for 15-20 minutes, and breakfast at 8.0 to 8.30 a.m. The early morning repetition lesson is an abomination which ought to be abolished. It is of little value, and usually means that the sleeping time is curtailed.

### Breakfast.

Breakfast should consist of two courses. First, some cereal food, such as porridge, quaker oats, etc., with salt, milk, cream or golden syrup; or bread and milk. Second, eggs in some form, fish, bacon, ham, tongue, meat rissoles, etc.; bread, rolls, toast and butter; cocoa, milk or weak tea to drink; fruit, lettuce, radishes, etc., when in season, and sufficiently inexpensive. The buttery system works satisfactorily in large schoolhouses. Under this system the boy selects his second course from the daily menu, and thus is certain to get what he likes and his proper share.

In the middle of the morning milk and biscuit, or fruit, should be given. This is especially necessary during cold weather, and for delicate boys.

### The Dinner Menu.

At dinner the first course should consist of meat, potatoes and green vegetables, the portion for each boy being put on his plate by the carver. Fish may be given once a week, preceded by a vegetable purèe. Any kind of simple pudding is suitable for a second course, especially suet puddings and those containing jam, marmalade, etc.; or shapes with stewed fruit, prunes, or jam. If the boy is still hungry let him have plain cheese, bread and butter. Cheese is a valuable addition to the diet. It should not be an American cheese, or made from skimmed milk, unless the diet is liberal in fat. It should neither be too new nor much decayed. It should only be allowed at the midday meal or at lunch.

### Tea.

Tea should be given at 6.0 p.m., and consist of unlimited bread and butter, various kinds of jams, plain cakes, freshly potted meat, fish, egg, cress or tomato sandwiches, plain cakes and biscuits; milk, cocoa, or weak tea. It should be sufficiently liberal to make supper unnecessary. Older boys, who do not go to bed until 10.0 or 10.30 p.m., should have more proteid food at tea, such as meat rissoles, calves' head, eggs, etc. If not, milk and biscuit must be allowed at 9.0 p.m. The late meal of bread, beer and cheese should not be allowed. Alcohol in all forms is unnecessary during school life. Water, barley water, oatmeal water or lemonade should be drunk at dinner. Such drinks should always be at hand in the house and playing fields. Small, young and delicate boys should be served first, so that they get good food, and have plenty of time for eating. Nitrogenous foods and milk are of the utmost importance. Animal proteids must not be replaced by vegetable ones. A boy requires from one-half to one pound of nitrogenous food daily, and a pint to a pint and a half of milk. The milk supply must be under careful supervision. Clear soups, beef tea, and meat extracts should not be given, for they are stimulants rather than foods, and spoil the child's appetite for simpler and more valuable foods. The only tinned foods permissible are sardines.

Refusal of food indicates a visit to the tuck shop, a hamper from home, illness, sameness of diet, tainted food, or some neglect in the service of meals. Girls require just as liberal a diet as boys, if they get as much exercise. In the best girls' schools the exercise is as liberal as in boys' schools. The period of rapid growth takes place two years earlier in girls than in boys, namely, at 11 to 16 years of age. At this period it is most essential that food and exercise should be sufficient for the perfect development, physically and mentally, of the girl and future mother.

